# Supplementary material for: Host-Strain-Specific Responses to Pneumonia Virus of Mice Infection: A Study of Lesions, Viral Load, and Cytokine Expression
Source: Viruses. 2025 Apr 9;17(4):548. doi: 10.3390/v17040548 (PMC12031304; doi:10.3390/v17040548)
Supplement: Supplementary file 1 [file viruses-17-00548-s001.zip › viruses-3522701-supplementary.pdf]

SUPPLEMENTAL MATERIAL

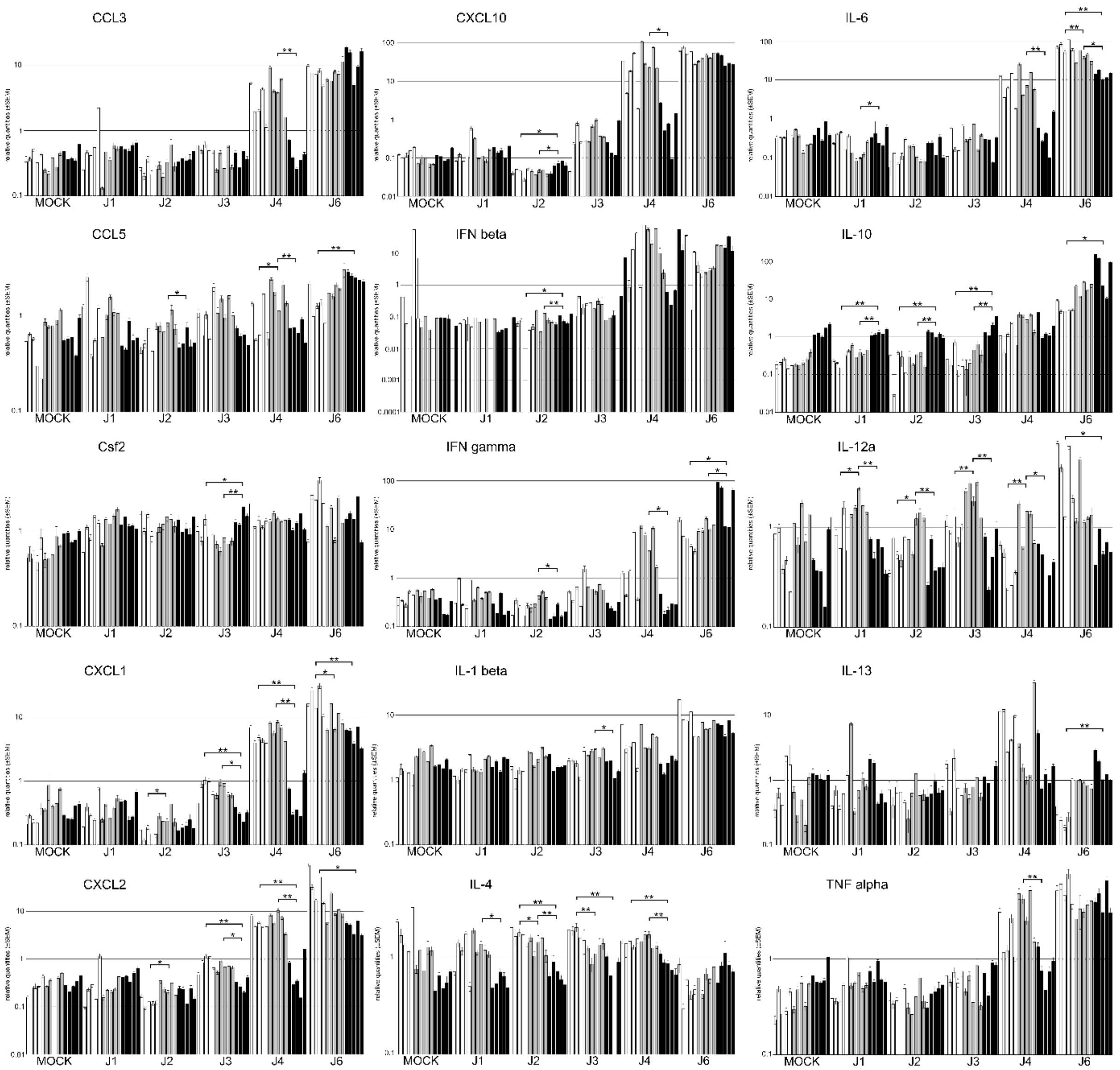

Figure S1. Logarithmic Scale Representation of Relative Cytokine Expression (qRT-PCR) at 1–4 and 6 d.p.i. Each bar = one individual. White bars = 129/Sv mice, gray bars = BALB/c mice, and black bars = SJL/J mice. Error bars = standard error of the mean. The statistical significance of differences between lines was calculated for each cytokine at each time point and is indicated above the corresponding histogram bars (* = $p \leq 0.05$, **= $p \leq 0.01$).

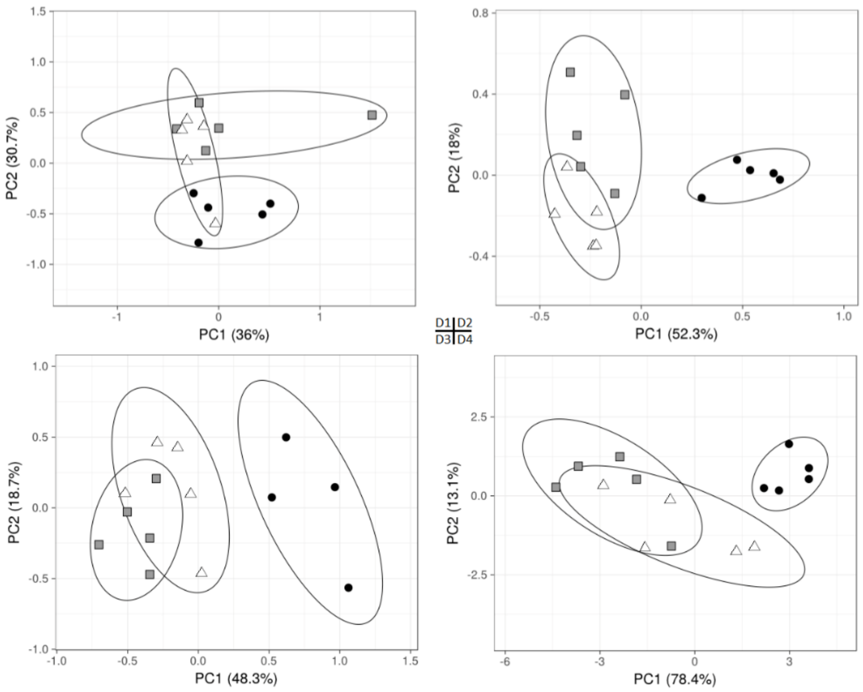

Figure S2. PCA scatterplots at 1-4 d.p.i., each dot represents 1 mouse, shape- and color-coded by inbred line. White triangles = 129/Sv, grey squares = BALB/c, and black circles = SJL/J. Prediction ellipses are such that with a probability of 0.85, a new observation from the same group will fall inside the ellipse. N = 15 data points, except for day 3p.i. when N = 14.

Table S1. Correlation analyses between microscopic lesion score (Micro.), pulmonary viral load (V. load), and 15 cytokines in 129/Sv mice (n=30). The values in the lower left half of the table correspond to Pearson's r and the values in upper right half of the table correspond to the associated p-values (after Bonferroni correction).

129/Sv	micro	viral load	IFNβ	CCL3	CCL5	CXCL1	CXCL10	CXCL2	Csf2	IL-6	IL-1β	TNF	IFNγ	IL-10	IL-12a	IL-13	IL-4
micro		4.0×10^{-08}	9.8×10^{-04}	2.6×10^{-07}	3.4×10^{-01}	2.4×10^{-06}	3.9×10^{-07}	2.6×10^{-04}	4.7×10^{-03}	2.2×10^{-06}	1.8×10^{-05}	4.6×10^{-07}	9.4×10^{-05}	2.5×10^{-06}	2.5×10^{-03}	4.3×10^{-01}	3.5×10^{-03}
viral load	0.86		4.5×10^{-04}	3.3×10^{-09}	2.0×10^{-01}	1.5×10^{-06}	5.2×10^{-09}	2.6×10^{-03}	5.4×10^{-04}	1.7×10^{-06}	4.9×10^{-04}	5.8×10^{-08}	1.7×10^{-04}	3.3×10^{-06}	1.4×10^{-02}	5.1×10^{-01}	7.5×10^{-04}
IFNβ	0.62	0.65		7.0×10^{-05}	2.1×10^{-02}	4.7×10^{-02}	4.2×10^{-05}	3.8×10^{-04}	8.7×10^{-01}	1.8×10^{-02}	3.5×10^{-03}	8.1×10^{-03}	7.1×10^{-11}	4.8×10^{-06}	3.4×10^{-02}	8.8×10^{-01}	7.1×10^{-02}
CCL3	0.83	0.89	0.71		3.8×10^{-02}	1.9×10^{-09}	3.3×10^{-13}	1.3×10^{-07}	8.8×10^{-04}	5.9×10^{-10}	1.1×10^{-05}	2.7×10^{-13}	1.1×10^{-07}	9.1×10^{-11}	7.6×10^{-06}	8.5×10^{-01}	7.3×10^{-05}
CCL5	0.2	0.26	0.46	0.42		2.1×10^{-01}	5.5×10^{-02}	3.5×10^{-02}	8.6×10^{-01}	1.5×10^{-01}	1.5×10^{-01}	1.2×10^{-01}	5.3×10^{-03}	4.6×10^{-02}	5.3×10^{-02}	6.6×10^{-01}	1.2×10^{-01}
CXCL1	0.79	0.8	0.4	0.89	0.26		5.4×10^{-09}	8.0×10^{-06}	2.3×10^{-06}	9.5×10^{-16}	1.4×10^{-04}	9.6×10^{-16}	5.7×10^{-04}	2.1×10^{-06}	5.7×10^{-07}	7.9×10^{-01}	8.4×10^{-05}
CXCL10	0.82	0.88	0.72	0.95	0.39	0.88		1.6×10^{-05}	1.3×10^{-03}	1.4×10^{-08}	2.6×10^{-04}	5.0×10^{-11}	8.5×10^{-08}	4.1×10^{-08}	1.4×10^{-04}	9.6×10^{-01}	3.3×10^{-04}
CXCL2	0.67	0.58	0.65	0.84	0.42	0.77	0.75		8.5×10^{-02}	1.1×10^{-06}	2.1×10^{-06}	4.6×10^{-06}	6.4×10^{-08}	5.1×10^{-10}	1.3×10^{-11}	5.7×10^{-01}	7.4×10^{-03}
Csf2	0.55	0.64	0.04	0.62	0.04	0.79	0.61	0.35		1.7×10^{-05}	5.4×10^{-02}	1.4×10^{-05}	3.2×10^{-01}	2.5×10^{-02}	1.7×10^{-02}	9.0×10^{-01}	1.8×10^{-02}
IL-6	0.79	0.8	0.47	0.9	0.29	0.97	0.87	0.81	0.75		2.8×10^{-04}	1.6×10^{-15}	1.0×10^{-04}	8.4×10^{-09}	5.5×10^{-08}	4.0×10^{-01}	2.2×10^{-05}
IL-1β	0.75	0.65	0.56	0.76	0.3	0.69	0.67	0.79	0.39	0.67		1.1×10^{-04}	1.6×10^{-04}	1.9×10^{-05}	2.4×10^{-04}	2.1×10^{-01}	2.7×10^{-02}
TNF	0.83	0.86	0.53	0.96	0.32	0.97	0.93	0.79	0.76	0.97	0.71		6.2×10^{-05}	7.3×10^{-08}	2.5×10^{-06}	8.5×10^{-01}	7.6×10^{-05}
IFNγ	0.7	0.68	0.92	0.84	0.54	0.64	0.85	0.85	0.21	0.7	0.69	0.72		3.2×10^{-10}	7.8×10^{-05}	5.5×10^{-01}	7.1×10^{-03}
IL-10	0.79	0.79	0.78	0.92	0.4	0.79	0.86	0.91	0.45	0.88	0.75	0.86	0.91		7.1×10^{-07}	4.6×10^{-01}	4.0×10^{-04}
IL-12a	0.58	0.49	0.42	0.77	0.39	0.82	0.69	0.93	0.47	0.85	0.67	0.8	0.71	0.81		2.0×10^{-01}	4.9×10^{-03}
IL-13	0.17	0.14	−0.03	0.04	−0.09	−0.06	−0.01	−0.12	0.03	−0.18	0.26	−0.04	−0.13	−0.16	−0.26		4.4×10^{-01}
IL-4	−0.56	−0.63	−0.37	−0.71	−0.32	−0.70	−0.66	−0.52	−0.47	−0.74	−0.44	−0.72	−0.52	−0.65	−0.54	0.16	

Table S2. Correlation analyses between microscopic lesion score (Micro.), pulmonary viral load (V. load), and 15 cytokines in BALB/c mice (n=30). The values in the lower left half of the table correspond to Pearson's r and the values in upper right half of the table correspond to the associated p-values (after Bonferroni correction).

BALB/c	micro	viral load	IFNβ	CCL3	CCL5	CXCL1	CXCL10	CXCL2	Csf2	IL-6	IL-1β	TNF	IFNγ	IL-10	IL-12a	IL-13	IL-4
micro		1.4×10^{-04}	1	3.2×10^{-04}	3.5×10^{-02}	2.4×10^{-04}	1.1×10^{-02}	3.6×10^{-03}	1	1.3×10^{-02}	4.0×10^{-04}	1.7×10^{-03}	4.0×10^{-03}	6.1×10^{-02}	1	1	1
viral load	0.81		2.5×10^{-01}	2.6×10^{-06}	4.9×10^{-04}	1.0×10^{-05}	8.9×10^{-07}	9.4×10^{-04}	1	1.6×10^{-04}	4.1×10^{-05}	1.6×10^{-09}	3.6×10^{-08}	2.8×10^{-02}	1	1	1
IFNβ	0.33	0.59		1	1	1	1.2×10^{-03}	1	1	1	1	3.2×10^{-03}	1	1	1	1	1
CCL3	0.79	0.87	0.49		2.6×10^{-07}	1.3×10^{-04}	1.9×10^{-07}	3.7×10^{-03}	1	6.6×10^{-05}	5.3×10^{-08}	7.6×10^{-08}	2.3×10^{-11}	1.0×10^{-04}	1	1	1
CCL5	0.67	0.78	0.48	0.89		8.6×10^{-02}	1.4×10^{-04}	5.1×10^{-01}	1	5.8×10^{-02}	1.6×10^{-04}	2.9×10^{-05}	1.9×10^{-05}	1.6×10^{-02}	1	1	1
CXCL1	0.8	0.85	0.38	0.81	0.64		3.2×10^{-03}	1.6×10^{-14}	8.8×10^{-01}	2.5×10^{-08}	7.0×10^{-06}	5.5×10^{-05}	2.6×10^{-04}	8.4×10^{-02}	1	1	1
CXCL10	0.71	0.88	0.76	0.9	0.81	0.74		4.4×10^{-02}	1	2.0×10^{-02}	4.3×10^{-05}	4.5×10^{-09}	1.7×10^{-06}	8.1×10^{-01}	1	1	1
CXCL2	0.74	0.77	0.3	0.74	0.56	0.98	0.66		1	1.3×10^{-07}	7.5×10^{-05}	3.0×10^{-03}	2.9×10^{-03}	2.2×10^{-01}	1	1	1
Csf2	0.23	0.29	0.17	0.32	0.18	0.53	0.27	0.47		1	1	1	1	1	1	1	1
IL-6	0.7	0.81	0.16	0.82	0.65	0.91	0.69	0.9	0.44		7.0 × 10−07	8.4 × 10−03	1.5×10^{-05}	1.4×10^{-04}	1	1	2.4×10^{-02}
IL-1β	0.79	0.83	0.36	0.91	0.81	0.86	0.83	0.82	0.32	0.88		2.1 × 10−04	3.2×10^{-06}	3.3×10^{-03}	1	1	8.8×10^{-01}
TNF	0.76	0.93	0.74	0.9	0.83	0.82	0.93	0.74	0.39	0.71	0.8		1.6×10^{-07}	2.5×10^{-01}	1	1	1
IFNγ	0.73	0.91	0.49	0.95	0.84	0.8	0.87	0.74	0.26	0.84	0.87	0.9		1.6×10^{-05}	1	1	1
IL-10	0.65	0.68	−0.01	0.81	0.69	0.64	0.53	0.6	0.17	0.81	0.74	0.59	0.84		1	1	5.8×10^{-02}
IL-12a	0.09	0.09	−0.15	0.01	0.12	0.32	0.01	0.44	−0.03	0.33	0.2	0.02	0.02	−0.02		1	1
IL-13	0.17	0.03	0.06	−0.05	−0.03	0.03	0.07	−0.02	0.01	−0.06	0.09	0	−0.08	−0.11	−0.22		1
IL-4	−0.37	−0.43	0.28	−0.43	−0.38	−0.47	−0.23	−0.48	−0.23	−0.68	−0.53	−0.23	−0.46	−0.65	−0.35	0.17	

Table S3. Correlation analyses between microscopic lesion score (Micro.), pulmonary viral load (V. load), and 15 cytokines in SJL/J mice (n=29). The values in the lower left half of the table correspond to Pearson's r and the values in upper right half of the table correspond to the associated p-values (after Bonferroni correction).

SJL/J	Micro.	V. load	IFNβ	CCL3	CCL5	CXCL 1	CXCL 10	CXCL 2	Csf2	IL-6	IL-1β	TNFα	IFNγ	IL-10	IL-12a	IL-13	IL-4
Micro.		2.1×10^{-02}	1	6.2×10^{-01}	7.5×10^{-01}	1.4×10^{-01}	1.3×10^{-01}	2.3×10^{-01}	1	1.6×10^{-01}	4.8×10^{-01}	1.6×10^{-01}	8.6×10^{-01}	6.5×10^{-01}	1	1	1
V. load	0.7		5.1×10^{-06}	5.6×10^{-03}	9.7×10^{-06}	1.5×10^{-09}	8.1×10^{-06}	2.7×10^{-09}	1	2.4×10^{-05}	2.4×10^{-06}	7.7×10^{-08}	1.4×10^{-01}	1.4×10^{-01}	1	1	1
IFNβ	0.54	0.89		2.4×10^{-03}	4.6×10^{-06}	1.6×10^{-10}	7.4×10^{-05}	1.8×10^{-10}	8.0×10^{-02}	5.9×10^{-05}	7.5×10^{-10}	2.3×10^{-14}	3.5×10^{-01}	5.7×10^{-01}	1	1	1
CCL3	0.56	0.74	0.78		8.3×10^{-08}	2.1×10^{-06}	3.0×10^{-11}	1.6×10^{-06}	1	6.2×10^{-12}	4.9×10^{-07}	1.0×10^{-04}	4.1×10^{-12}	2.3×10^{-10}	1	1	1
CCL5	0.55	0.86	0.89	0.91		2.6×10^{-09}	2.3×10^{-11}	7.8×10^{-09}	1	4.3×10^{-11}	7.2×10^{-09}	1.8×10^{-07}	9.2×10^{-05}	1.7×10^{-04}	1	1	1
CXCL 1	0.63	0.94	0.96	0.88	0.94		2.3×10^{-10}	2.1×10^{-24}	1	5.1×10^{-09}	3.7×10^{-14}	1.3×10^{-13}	1.8×10^{-03}	2.8×10^{-03}	1	1	1
CXCL 10	0.63	0.86	0.85	0.96	0.96	0.95		8.8×10^{-10}	1	2.2×10^{-12}	2.9×10^{-09}	1.5×10^{-06}	6.3×10^{-08}	7.9×10^{-08}	1	1	1
CXCL 2	0.61	0.94	0.96	0.88	0.93	1	0.94		1	7.1×10^{-09}	8.2×10^{-14}	2.6×10^{-13}	1.8×10^{-03}	3.2×10^{-03}	1	1	1
Csf2	0.43	0.45	0.67	0.15	0.3	0.46	0.28	0.44		1	1	7.5×10^{-01}	1	1	1	1	1
IL-6	0.62	0.85	0.85	0.96	0.96	0.93	0.97	0.93	0.23		6.0×10^{-09}	4.6×10^{-07}	8.1×10^{-07}	2.8×10^{-06}	1	1	1
IL-1β	0.57	0.88	0.95	0.9	0.93	0.98	0.94	0.98	0.44	0.93		2.3×10^{-10}	1.0×10^{-03}	2.3×10^{-03}	1	1	1
TNFα	0.62	0.91	0.98	0.82	0.9	0.97	0.88	0.97	0.55	0.9	0.95		4.6×10^{-02}	7.9×10^{-02}	1	1	1
IFNγ	0.54	0.63	0.61	0.96	0.83	0.77	0.91	0.77	0.01	0.89	0.78	0.67		4.6×10^{-24}	1	1	1
IL-10	0.56	0.63	0.59	0.95	0.82	0.75	0.91	0.75	0.01	0.88	0.76	0.65	1		1	1	1
IL-12a	0.45	0.28	0.21	0.19	0.19	0.24	0.22	0.24	0.19	0.25	0.15	0.33	0.14	0.15		1	1
IL-13	0.32	0.29	0.17	0.24	0.19	0.25	0.28	0.26	0.19	0.2	0.15	0.24	0.27	0.29	0.26		1
IL-4	0.31	0.3	0.19	−0.02	0.22	0.12	0.08	0.12	0.17	0.11	0.05	0.21	−0.09	−0.08	0.48	0.27	
